# Role of Libraries in Human Flourishing: Adolescents’ Motivational Orientation for Occupation

**DOI:** 10.3390/ijerph182111209

**Published:** 2021-10-25

**Authors:** Yusuke Matsuyama, Takeo Fujiwara

**Affiliations:** Department of Global Health Promotion, Tokyo Medical and Dental University, Bunkyo, Tokyo 113-8510, Japan; fujiwara.hlth@tmd.ac.jp

**Keywords:** flourishing, adolescent, intrinsic motivation, community

## Abstract

Introduction: Adolescence is crucial for human flourishing and strongly influences having meaning in life. We investigated the association between local public library density as a shared resource and motivational orientation toward their occupation in Japanese adolescents. Methods: A longitudinal study was conducted using data from a nationwide birth cohort survey in Japan (*n* = 12,184). At age 7, their caregivers answered questionnaires on children including the number of books read. Library density (low, moderate, or high) in each municipality was obtained from national statistics. At age 15, the adolescents indicated whether they had decided on an occupation and selected motivational orientations from among intrinsic (own ability and interest), extrinsic (high earnings, social class, or job stability), and altruistic (social contribution) orientations. Multilevel linear probability models were fitted, adjusting for confounders, including household socioeconomic status and city size. Results: Intrinsic, extrinsic, and altruistic motivations for desired occupation were reported by 40.7%, 31.9% and 41.8% of participants, respectively. Living in a municipality with a high library density at age 7 was associated with having intrinsic motivation at age 15 than low density by 3.1 percentage points (95% confidence interval (CI): 0.35, 5.85). The association was more prominent for those with lower income (*P* for interaction = 0.026). Neither extrinsic nor altruistic motivations were associated with library density (coefficient: −0.13; 95% CI: −2.81, 2.56; coefficient: 0.08; 95% CI: −2.72, 2.88 percentage points, respectively). Conclusions: Developing libraries in communities could encourage intrinsic motivation in adolescents, specifically for those in low-income households.

## 1. Introduction

Having meaning in life is a central part of human flourishing [1]; such meaning has three components: a sense of a core goal (i.e., purpose), a sense of the inherent value of life (i.e., significance), and a comprehensive understanding of the self and the world (i.e., coherence) [2]. Youth is a crucial period for forming a sense of meaning [3], and benefits of having meaning in life for health outcomes are reported throughout life. Having meaning in life is associated with psychological well-being in adolescents [4], with lower depressive symptoms in young adults [5], and with delayed cognitive impairment in older people [6]. As a result of these observations, meaning in life has become a potential target for public health intervention [1,5,7].

Occupation remains a dominant part of life in adulthood. People experience a sense of meaning when they are intrinsically motivated to pursue their occupation [8,9]. More specifically, people achieve a sense of meaning in their occupation when they can develop their inner selves, express their full potential, feel united with others, and serve others [8,9]. Intrinsic motivation in one’s occupation could lead to favorable social outcomes, such as positive career development [10] and early promotion [11]. Adolescence is a life-transition period that shapes the career in later life. Adolescents can improve their future well-being and social outcomes by making their career choice from intrinsic motivation. Cognitively stimulating home environments improve intrinsic academic motivation [12]; this implies that certain environments would encourage intrinsic motivation more than others. However, there is little research on the modifiable factors that could positively influence adolescents’ intrinsic motivation for their future careers.

Public libraries provide free access to information, reading materials, and a gathering space [13]. Exposure to various sources of information can help children find areas of interest and choose career paths. Reading books, which allows children to increase their knowledge and understanding of themselves and their world, might be associated with the later development of intrinsic motivation for an occupation due to their contribution to cognitive development and reading comprehension skills [14], which in turn would result in better academic performance [15] and increased motivation for the career. Additionally, some books may promote children’s creativity and broaden their perspectives. Xu and Pan reported that differences in life experience mediate the association between high socioeconomic status and creativity among university students. They suggested that this gap could be reduced by utilizing shared resources [16]. Further, social events held at public libraries can provide opportunities for children to be exposed to different experiences. This suggests that community public libraries work as shared resources available to adolescents and provide more than books for reading, acting as beneficial facilities for adolescents’ motivation. In this study, we hypothesized that libraries in the community contribute to the development of adolescents’ intrinsic motivation for their desired occupation and investigated it by utilizing a nationwide birth cohort study in Japan.

## 2. Materials & Methods

### 2.1. Study Participants

We analyzed data from the Longitudinal Survey of Newborns in the 21st Century, a nationwide birth cohort study in Japan. Details on the survey are reported elsewhere [17]. In brief, all infants born in Japan during the periods 10–17 January and 10–17 July in 2001 were recruited to participate using birth registration records (*n* = 53,575). Their caregivers answered mailed questionnaires when the babies were 6 months old (*n* = 47,010; response rate = 87.7%); after this, follow-up surveys were conducted annually. Caregivers answered the surveys from the first to the eleventh wave (i.e., when the children were aged from 0.5 to 11 years old), and both caregivers and children answered from the twelfth survey onwards (i.e., the children were aged 12 years or older). The present study used data from the seventh survey, conducted in 2008 (i.e., at 7 years old), and the 15th survey, conducted in 2016 (i.e., at 15 years old).

Figure S1 shows the flowchart for the survey responses. In the seventh survey, 40,598 caregivers received the questionnaire, and 36,978 responded (response rate: 90.6%). From the respondents in this group, 28,337 provided responses to the fifteenth survey (follow-up rate: 77.0% from the seventh wave and 61.3% from the first wave). Those children in the fifteenth wave who had not decided a desired occupation (*n* = 15,879) and who had not provided information on their municipalities of residence (*n* = 56), the number of books read (*n* = 123), or their father’s age (*n* = 100) were excluded, with data from 12,184 respondents remaining for the analysis.

### 2.2. Dependent Variable: Motivational Orientation for the Desired Occupation

At the age of 15, the respondents were asked whether they had chosen a desired occupation. Those who had were further asked for a reason for this, choosing from the following options: “For its high earnings” (earnings), “For its good reputation and high social class” (reputation), “Because it suits my ability” (ability), “Because of my interests” (interest), “Because it contributes to society and others” (altruistic), “Because it is stable” (stability), and “To follow in my parents’ work” (parents’ work). The prevalence of each response and its tetrachoric correlation coefficients (i.e., the correlation coefficients for binary variables) are reported in Supplementary Table S1. We classified these variables into three categories: intrinsic motivation (ability and interest), extrinsic motivation (earnings, reputation, or stability), and altruistic motivation. The item to follow in parents’ work was not used because it was strongly correlated with parents’ self-employment status.

### 2.3. Independent Variables

Information at birth, age 7, and age 15 was included in the use of the longitudinal birth cohort data. Variables at birth were collected from birth registration records, namely, child’s gender, mother’s age, father’s age, and parents’ educational attainment. During the seventh survey, the following information was assessed by the questionnaire to the caregivers: the number of books the children read in the previous month (0, 1, 2–3, 4–7, 8–11, and ≥12; used as a continuous variable coded with a midpoint for each bracket), having older siblings, having younger siblings, and parents’ employment status. The data on library density in municipalities were obtained from the Social Education Survey conducted in the same year [18]. At 15 years old, variables regarding living with both parents and equivalent income were assessed. The size of the municipality of residence was also obtained, according to the administrative classification.

### 2.4. Statistical Analysis

Multilevel linear probability models with random intercepts (level 1, individual; level 2, municipality (i.e., cities, towns, and villages, defined based on population); and level 3, prefecture) were fitted to investigate the association of library density with intrinsic, extrinsic, and altruistic motivations for a desired occupation.

A null model (i.e., an intercept-only model) for each outcome was fitted to evaluate the intraclass correlation. Four models were constructed. The main exposure variable, library density, was first included to assess a univariate association with motivation (model 1). Then, variables at birth and age 7 but excluding the number of books read were added to control the confounding factors at age 7 or before (model 2). In model 2, we did not include the number of books at age 7 to evaluate the extent to which the variable of reading books, independent from other confounding variables, explains the association between library density and motivation. Therefore, we added the number of books reading in model 3. Finally, the variables at age 15 were added to control for factors related to motivation at age 15 (model 4). A stratification analysis according to equivalent income was also performed to investigate whether the association with library density differed by income strata. An effect modification by the lowest income group for the association between library density and intrinsic motivation was tested because the point estimate was larger in the lowest income than the other three categories. Missing information on covariates was coded with dummy variables and included in the analysis. Multilevel analysis was performed by MLwiN [19] with the *runmlwin* command [20] from Stata 16.1 software. Other analyses were performed using Stata 16.1 software.

## 3. Results

Table 1 presents the demographic characteristics of the respondents. Of the 12,184 respondents, 40.7%, 31.9% and 41.8% reported having intrinsic, extrinsic, and altruistic motivations for a desired occupation, respectively. Girls (*p* < 0.001), having parents with high education (*p* < 0.001), reading books (*p* < 0.001), living in municipalities with higher library densities (*p* = 0.003), not having older siblings (*p* < 0.001), and living with both parents (*p* = 0.005) were significantly associated with having intrinsic motivation. Extrinsic motivation was associated with boys (*p* < 0.001), having parents with higher education (*p* < 0.001), not having older siblings (*p* < 0.001), and having a higher equivalent income (*p* < 0.001). The following were positively associated with altruistic motivation: girls (*p* < 0.001), having parents with high education (*p* < 0.001), reading books (*p* < 0.001), not having older siblings (*p* = 0.002), having younger siblings (*p* = 0.028), having neither parent be self-employed (*p* = 0.002), and having a higher equivalent income (*p* < 0.001).

The null models showed little variation in prefecture- and municipality-level (Supplementary Table S2, with the intraclass correlations at level 2 and level 3 being 0.41% and 0.24% for intrinsic motivation, 0.62% and 0.11% for extrinsic motivation, and 0.09% and 0.59% for altruistic motivation).

**Figure 1:**
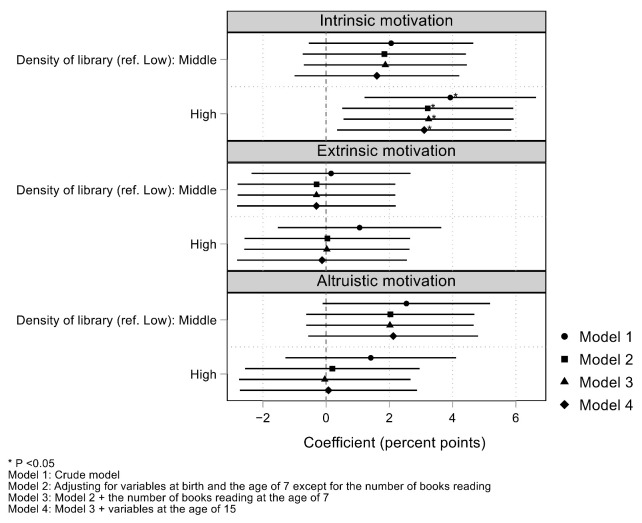
Association of libraries with motivational orientation for the desired occupation; multilevel linear probability model (level 1, individual: *n* = 12,184; level 2, municipality of residence wave 15: *n* = 1457; level 3, prefecture of residence wave 15: *n* = 47).

Figure 1 depicts the association of library density with motivational orientation for the desired occupation. Estimated numbers are also reported in Supplementary Table S2. High library density was significantly associated with intrinsic motivation (model 1; coefficient, 3.93; 95% confidence interval (CI), 1.21, 6.64). The association remained significant after adjusting for variables at birth and age 7 (model 2; coefficient, 3.21; 95% CI, 0.51, 5.92), but it was not explained by the number of books read (model 3; coefficient, 3.24; 95% CI, 0.55, 5.93). After adjusting for all covariates, high library density was significantly associated with intrinsic motivation than low library density by 3.10 percentage points (model 4; 95% CI, 0.35, 5.85). Extrinsic motivation was not associated with library density (coefficient, −0.13; 95% CI, −2.81, 2.56). Altruistic motivation was not associated with library density (coefficient, 0.08; 95% CI, −2.72, 2.88).

As reported in Supplementary Table S3, girls were more likely to have intrinsic (coefficient, 3.99; 95% CI, 2.23, 5.76) and altruistic (coefficient, 8.05; 95% CI, 6.28, 9.81) motivations than boys but less likely to have extrinsic motivation (coefficient, −6.55; 95% CI, −8.22, −4.88). Parents’ higher education was associated with every type of motivation (coefficient, 3.56; 95% CI, 0.67, 6.45 for intrinsic motivation; coefficient, 5.47; 95% CI, 2.73, 8.20 for extrinsic motivation; and coefficient, 5.21; 95% CI, 2.31, 8.10 for altruistic motivation), while the highest income was associated with extrinsic (coefficient, 6.79; 95% CI, 4.12, 9.47) and altruistic (coefficient, 2.90; 95% CI, 0.07, 5.73) motivation but not with intrinsic motivation (coefficient, −0.45; 95% CI, −3.27, 2.38). The number of books read was associated with intrinsic (coefficient, 0.62; 95% CI, 0.39, 0.84) and altruistic (coefficient, 0.57; 95% CI, 0.35, 0.80) motivation but not with extrinsic motivation (coefficient, 0.16; 95% CI, −0.05, 0.38).

**Figure 2:**
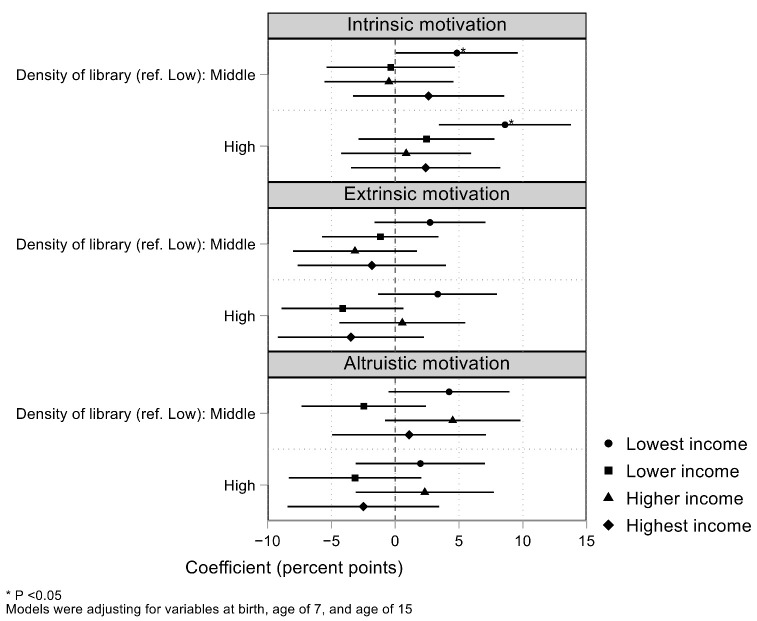
Stratification analysis by equivalent income showing the association of libraries with motivational orientation for the desired occupation.

**Figure 3:**
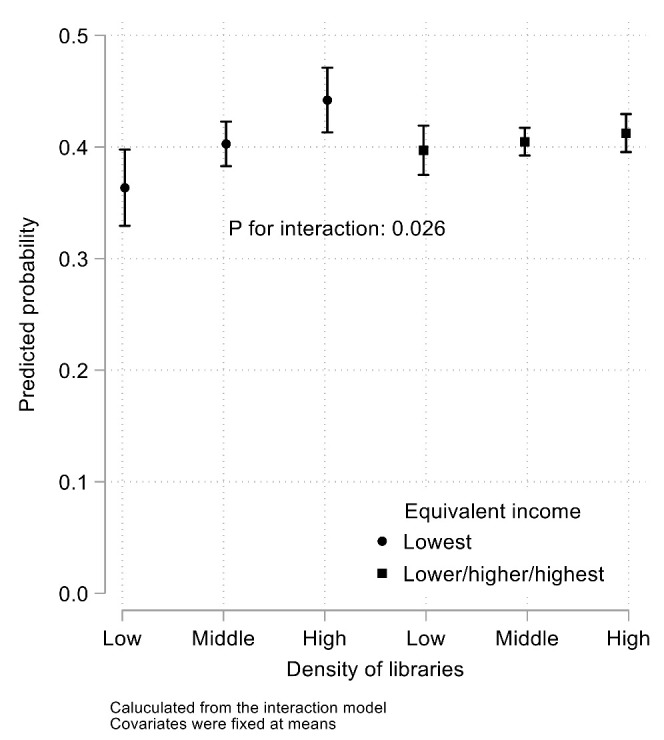
Predicted probability by a model with an interaction term.

Figure 2 and Supplementary Table S4 show the results of the stratification analysis by equivalent income. Library density was associated with intrinsic motivation among adolescents at the lowest income (coefficient, 8.60; 95% CI, 3.41, 13.78) but not at other income strata. The association was significantly more prominent in the lowest income category than in the other three categories combined (Figure 3, *P* for interaction: 0.026). No significant associations were observed between library density and extrinsic or altruistic motivation at any income stratum.

## 4. Discussion

This study found that library density in the municipality of residence at the age of 7 was positively associated with the possession of intrinsic motivation for an occupation desired at the age of 15 (coefficient, 3.10; 95% CI, 0.35, 5.85). The association was more prominent in adolescents from the lowest income backgrounds (*p* for interaction: 0.026). Extrinsic or altruistic motivations were not associated with library density.

A few previous studies reported factors that influence adolescents’ motivational orientation for their desired occupation. Hirschi (2010) reported that girls tended to report higher intrinsic and lower extrinsic values for work [10], which is in line with the present study. Another study reported an association between warm and acceptable parenting styles and adolescents’ career decision-making [21]. Psychological support from parents and teachers cultivates adolescents’ self-efficacy, and this leads to an expectation of the best possible outcome for future career development [22]. In our study, the number of books read in the last month at age 7 would reflect a warm and accepting parenting style; therefore, it was associated with intrinsic and extrinsic motivations. This study added to the literature that community-level factors such as public library density are associated with adolescents’ intrinsic motivation for their career choice. Our interaction analysis found that the benefits of public libraries could be more prominent among children in low-income households. This is likely because, for example, public libraries provide materials and places for study, and this may compensate for the deprivation of resources among some children.

Public libraries are community resources providing services such as free access to information, reading, and a gathering space [13]. These resources could also increase adolescents’ intrinsic motivation. Exposure to various sources of information can help children find areas of interest and thereby to choose career paths following an intrinsic motivation. The possession of occupational information is associated with showing a consistent career pattern from adolescence to early adulthood [23]. Having career role models is a significant predictor of planning a career path [23]. Children may find their role models from biographies. Social events held at public libraries provide opportunities for children to be exposed to different experiences. A high availability of books could develop children’s reading skills, which are associated with improved academic outcomes [24], which would in turn increase intrinsic motivation for the career. In this study, the association of public libraries with intrinsic motivation remained after an adjustment for the number of books read and other covariates. This suggests that public libraries provide more than books for reading, acting as beneficial facilities for adolescents’ intrinsic motivation.

An intrinsically motivated occupation can provide meaning in life [8,9]. However, the opportunity to work in this type of occupation is not equally distributed across social strata: those with lower socioeconomic backgrounds are less likely to have intrinsic motivation for an occupation [25]. Utilizing community resources is suggested to reduce the gap related to socioeconomic strata [16]. Our study showed that the benefit of public libraries was more prominent among adolescents with the lowest income category. Public libraries could be a potential intervention point for increasing the intrinsic motivation of adolescents, especially for those from lower socioeconomic backgrounds. Interestingly, parents’ higher education was associated with intrinsic motivation, whereas high income was not. Parents with higher education may prioritize values other than earnings in their job, which can lead to children’s intrinsic motivation for their job.

The present study has several limitations. First, the measurement of motivational orientation for the desired occupation is not yet validated. Second, the survey was self-reported by caregivers and adolescents. Thus, some self-reported biases, such as social desirability bias, exist. Moreover, cultural issues around the prestige of certain occupations might have influenced the adolescents’ response. For example, it was reported that people rated judges, professors, and physicians as having high prestige in Japan [26], although it could have changed in recent decades. However, the present study focused on adolescents’ motivations that drive them toward a future occupation regardless of cultural prestige. Third, information was not available on whether children used public libraries, while the observed association could be robust for response bias because data on public libraries were obtained from other national statistics. The association of public libraries with intrinsic motivation could differ by municipal characteristics, but our auxiliary analysis, stratified by city size, showed similar findings (Supplementary Table S5). Fourth, although we used nationwide birth cohort data, only 61.3% of the participants were followed until the age of 15. The caregivers who responded to the fifteenth survey were older and had higher educational attainment than those who were lost to follow-up. As reported in Table 1, children with higher parental education were more likely to report intrinsic, extrinsic, or altruistic motivations for the desired occupation. Thus, the prevalence of these motivations might be overestimated. Fifth, the pathways linking library density and intrinsic motivation were not empirically evaluated due to a lack of data. Sixth, while we adjusted for confounding factors within the available data, there could be residual confounding factors, such as parenting style. Moreover, it is unknown what type of books the adolescent read at the age of 7 or whether the book selection was parent-driven or child-driven. Further studies assessing these variables are required to assess the robustness of the finding.

## 5. Conclusions

This study, utilizing a Japanese nationwide birth cohort survey, found a positive association between library density in the municipality of residence at age 7 and intrinsic motivation for a desired occupation at age 15. Developing public libraries can help encourage intrinsic motivation for an occupation among adolescents. Notably, the benefit of public libraries might be more prominent among adolescents from lower socioeconomic backgrounds. Developing public libraries could reduce the gap in intrinsic motivation among adolescents by socioeconomic strata. It is informative to assess in future studies what kind of occupation the adolescents obtain. Moreover, the pathways between library density and intrinsic motivation need to be investigated. For example, the frequency of social events at libraries might be a candidate variable.

## Figures and Tables

**Table 1:** Demographic characteristics of the study respondents (*n* = 12,184).

	Total	Intrinsic Motivation			Extrinsic Motivation			Altruistic Motivation		
		No	Yes		No	Yes		No	Yes	
	*n* = 12184	59.3%	40.7%		68.1%	31.9%		58.2%	41.8%	
	*n* (column %) or Mean (SD)	Row % or Mean	Row % or Mean	*p*-Value	Row % or Mean	Row % or Mean	*p*-Value	Row % or Mean	Row % or Mean	*p*-Value
Variables at birth										
Child’s gender				<0.001			<0.001			<0.001
Boy	5397 (44.3%)	61.8%	38.2%		64.5%	35.5%		62.8%	37.2%	
Girl	6787 (55.7%)	57.3%	42.7%		71.0%	29.0%		54.4%	45.6%	
Mother’s age	30.5 (4.3)	30.5	30.6	0.200	30.5	30.6	0.270	30.4	30.6	0.050
Father’s age	32.6 (5.4)	32.6	32.6	0.610	32.6	32.7	0.350	32.5	32.7	0.023
Parent’s education				<0.001			<0.001			<0.001
Both college	1562 (12.8%)	55.2%	44.8%		62.7%	37.3%		53.6%	46.4%	
Either college	3623 (29.7%)	57.8%	42.2%		65.6%	34.4%		55.6%	44.4%	
Neither college	6764 (55.5%)	60.7%	39.3%		70.6%	29.4%		60.4%	39.6%	
Missing	235 (1.9%)	67.2%	32.8%		73.2%	26.8%		62.6%	37.4%	
Variables at age 7										
Number of books read in previous month				<0.001			0.210			<0.001
None	707 (5.8%)	66.6%	33.4%		68.3%	31.7%		61.8%	38.2%	
1	1344 (11.0%)	63.2%	36.8%		70.5%	29.5%		62.1%	37.9%	
2 or 3	3544 (29.1%)	60.2%	39.8%		69.0%	31.0%		60.1%	39.9%	
4–7	3193 (26.2%)	59.4%	40.6%		67.2%	32.8%		58.7%	41.3%	
8–11	1167 (9.6%)	57.4%	42.6%		67.2%	32.8%		56.0%	44.0%	
≥12	2229 (18.3%)	54.1%	45.9%		67.1%	32.9%		52.0%	48.0%	
Library density				0.003			0.860			0.240
Low	2224 (18.3%)	62.1%	37.9%		68.4%	31.6%		59.4%	40.6%	
Middle	4647 (38.1%)	59.7%	40.3%		68.4%	31.6%		57.0%	43.0%	
High	4868 (40.0%)	57.6%	42.4%		67.7%	32.3%		58.6%	41.4%	
Missing	445 (3.7%)	60.0%	40.0%		68.8%	31.2%		58.9%	41.1%	
Having older sibling				<0.001			<0.001			0.002
No	6136 (50.4%)	57.7%	42.3%		66.3%	33.7%		56.8%	43.2%	
Yes	6048 (49.6%)	60.9%	39.1%		70.0%	30.0%		59.6%	40.4%	
Having younger sibling				0.087			0.100			0.028
No	6508 (53.4%)	60.0%	40.0%		68.8%	31.2%		59.1%	40.9%	
Yes	5676 (46.6%)	58.5%	41.5%		67.4%	32.6%		57.1%	42.9%	
Self-employed parent				0.260			0.140			0.002
No	9740 (79.9%)	59.1%	40.9%		67.7%	32.3%		57.4%	42.6%	
Yes	1653 (13.6%)	59.2%	40.8%		70.0%	30.0%		60.7%	39.3%	
Missing	791 (6.5%)	62.1%	37.9%		69.3%	30.7%		62.2%	37.8%	
Variables at age 15										
Living with both parents				0.005			0.250			0.140
No	1555 (12.8%)	62.6%	37.4%		69.4%	30.6%		59.9%	40.1%	
Yes	10629 (87.2%)	58.8%	41.2%		67.9%	32.1%		57.9%	42.1%	
Equivalent income				0.320			<0.001			<0.001
Lowest	2953 (24.2%)	60.5%	39.5%		72.5%	27.5%		61.4%	38.6%	
Lower	2990 (24.5%)	59.8%	40.2%		68.7%	31.3%		58.3%	41.7%	
Higher	3085 (25.3%)	58.9%	41.1%		66.5%	33.5%		56.8%	43.2%	
Highest	2821 (23.2%)	57.9%	42.1%		63.8%	36.2%		56.2%	43.8%	
Missing	335 (2.7%)	59.4%	40.6%		76.7%	23.3%		57.6%	42.4%	
Size of residential city				0.270			0.150			0.720
Major city	3073 (25.2%)	58.5%	41.5%		67.2%	32.8%		58.3%	41.7%	
Middle city	2885 (23.7%)	59.0%	41.0%		67.1%	32.9%		57.3%	42.7%	
Small city	5112 (42.0%)	59.4%	40.6%		69.2%	30.8%		58.6%	41.4%	
Town	1114 (9.1%)	61.8%	38.2%		68.3%	31.7%		58.0%	42.0%	

Abbreviations: standard deviation, SD. *p*-values for mother’s age and father’s age were obtained by *t*-tests, and *p*-values for other variables were obtained by chi-square tests.

## Data Availability

The data that support the findings of this study are available from the authors upon reasonable request and with permission of The Japanese Ministry of Health, Labour and Welfare.

## Supplementary Materials

The following are available online at https://www.mdpi.com/article/10.3390/ijerph182111209/s1, Table S1: Prevalence and tetrachoric correlation coefficients among reasons for desired occupation, Table S2: Association of library density with the motivational orientation for the desired occupation; multilevel linear regression model, Table S3: Association between libraries and motivational orientation for the desired occupation; multilevel linear regression model, Table S4: Stratification analysis by equivalent income, Table S5: Stratification analysis by size of municipality of residence at age 15, Figure S1: Flowchart for respondent analysis
