# Supplementary material for: Role of Libraries in Human Flourishing: Adolescents’ Motivational Orientation for Occupation
Source: Int J Environ Res Public Health. 2021 Oct 25;18(21):11209. doi: 10.3390/ijerph182111209 (PMC8583462; doi:10.3390/ijerph182111209)

Supplementary Table S1. Prevalence and tetrachoric correlation coefficients among reasons for desired occupation (n = 12,184).

		Tetrachoric correlation coefficients						
	Prevalence	1	2	3	4	5	6	7
1. Income	23.3%	1.000						
2. Reputation	6.4%	0.569*	1.000					
3. Ability	47.7%	−0.122*	0.098*	1.000				
4. Interest	78.3%	−0.400*	−0.083*	0.289*	1.000			
5. Altruistic	41.8%	0.232*	0.184*	−0.006	−0.227*	1.000		
6. Stability	13.2%	0.448*	0.245*	−0.144*	−0.418*	0.273*	1.000	
7. Parents' work	3.3%	0.031	0.049	−0.207*	−0.352*	−0.025	0.054	1.000

*: P <0.05

Supplementary Table S2. Association of the library density with the motivational orientation for the desired occupation; multilevel linear regression model (level 1, individual: n = 12184; level 2, municipality of residence wave 15: n = 1457; level 3, prefecture of residence wave 15: n = 47).

	Null model	Model 1	Model 2	Model 3	Model 4
	Coef. (95% CI)	Coef. (95% CI)	Coef. (95% CI)	Coef. (95% CI)	Coef. (95% CI)
**Intrinsic motivation**					
Fixed effects					
Library density					
Low		ref.	ref.	ref.	ref.
Middle		2.05 (−0.55, 4.66)	1.84 (−0.74, 4.42)	1.87 (−0.71, 4.45)	1.60 (−1.00, 4.21)
High		3.93 (1.21, 6.64)	3.21 (0.51, 5.92)	3.24 (0.55, 5.93)	3.10 (0.35, 5.85)
Random effects					
Prefecture-level variance	0.06 (−0.01, 0.13)	0.04 (−0.02, 0.10)	0.04 (−0.02, 0.09)	0.03 (−0.02, 0.09)	0.03 (−0.02, 0.09)
City-level variance	0.10 (−0.06, 0.26)	0.08 (−0.08, 0.23)	0.06 (−0.09, 0.21)	0.06 (−0.09, 0.21)	0.05 (−0.09, 0.20)
Individual-level variance	23.98 (23.36, 24.60)	23.99 (23.37, 24.61)	23.89 (23.27, 24.50)	23.83 (23.21, 24.44)	23.82 (23.21, 24.44)
**Extrinsic motivation**					
Fixed effects					
Library density					
Low		ref.	ref.	ref.	ref.
Middle		0.16 (−2.36, 2.67)	−0.30 (−2.80, 2.19)	−0.31 (−2.80, 2.19)	−0.31 (−2.82, 2.20)
High		1.06 (−1.53, 3.64)	0.04 (−2.58, 2.66)	0.02 (−2.59, 2.64)	−0.13 (−2.81, 2.56)
Random effects					
Prefecture-level variance	0.02 (−0.02, 0.07)	0.03 (−0.02, 0.07)	0.04 (−0.01, 0.10)	0.04 (−0.01, 0.09)	0.05 (−0.01, 0.11)
City-level variance	0.13 (−0.02, 0.28)	0.13 (−0.02, 0.28)	0.10 (−0.04, 0.25)	0.10 (−0.04, 0.25)	0.08 (−0.06, 0.22)
Individual-level variance	21.56 (21.00, 22.11)	21.55 (21.00, 22.11)	21.33 (20.78, 21.88)	21.33 (20.78, 21.88)	21.27 (20.72, 21.82)
**Altruistic motivation**					
Fixed effects					
Library density					
Low		ref.	ref.	ref.	ref.
Middle		2.54 (−0.11, 5.18)	2.03 (−0.63, 4.69)	2.02 (−0.63, 4.67)	2.12 (−0.56, 4.81)
High		1.41 (−1.29, 4.11)	0.20 (−2.56, 2.96)	−0.04 (−2.75, 2.67)	0.08 (−2.72, 2.88)
Random effects					
Prefecture-level variance	0.02 (−0.03, 0.07)	0.02 (−0.03, 0.06)	0.03 (−0.02, 0.09)	0.02 (−0.03, 0.06)	0.02 (−0.03, 0.06)
City-level variance	0.14 (−0.02, 0.31)	0.14 (−0.02, 0.31)	0.15 (−0.01, 0.32)	0.17 (−0.00, 0.34)	0.17 (−0.00, 0.33)
Individual-level variance	24.17 (23.54, 24.79)	24.16 (23.54, 24.79)	23.83 (23.22, 24.45)	23.78 (23.17, 24.40)	23.77 (23.15, 24.38)

Abbreviations: coefficient, Coef; confidence interval, CI
Null model: intercept-only model
Model 1: library density was added
Model 2: model 1 + variables at age 7 except for the number of books reading (i.e., having older siblings, having younger siblings, and either self-employment)
Model 3: model 2 + the number of books reading at age 7
Model 4: model 3 + variables at age 15 (i.e., living with both parents, equivalent income, size of residential city)
Missing categories for covariates were included in the analysis but not reported to reduce the space

Supplementary Table S3. Association between libraries and motivational orientation for the desired occupation; multilevel linear regression model (level 1, individual: n = 12184; level 2, municipality of residence wave 15: n = 1457; level 3, prefecture of residence wave 15: n = 47).

	Intrinsic motivation<br>Coef. (95% CI)	Extrinsic motivation<br>Coef. (95% CI)	Altruistic motivation<br>Coef. (95% CI)
**Fixed effects**			
Library density at age 7			
Low	ref.	ref.	ref.
Middle	1.60 (−1.00, 4.21)	−0.31 (−2.82, 2.20)	2.12 (−0.56, 4.81)
High	3.10 (0.35, 5.85)	−0.13 (−2.81, 2.56)	0.08 (−2.72, 2.88)
Gender			
Boys	ref.	ref.	ref.
Girls	3.99 (2.23, 5.76)	−6.55 (−8.22, −4.88)	8.05 (6.28, 9.81)
Mother’s age	0.19 (−0.11, 0.48)	0.06 (−0.22, 0.34)	0.09 (−0.21, 0.38)
Father’s age	−0.05 (−0.27, 0.18)	0.03 (−0.19, 0.24)	0.18 (−0.05, 0.41)
Parents' education			
Neither college	ref.	ref.	ref.
Either college	1.78 (−0.31, 3.86)	3.55 (1.58, 5.53)	3.61 (1.52, 5.70)
Both college	3.56 (0.67, 6.45)	5.47 (2.73, 8.20)	5.21 (2.31, 8.10)
Having older siblings at age 7			
Yes	−3.11 (−5.22, −0.99)	−3.32 (−5.32, −1.32)	−2.09 (−4.20, 0.03)
Having younger siblings at age 7			
Yes	−0.15 (−2.35, 2.04)	0.71 (−1.37, 2.78)	1.55 (−0.65, 3.75)
Self-employed parent at age 7			
Yes	0.60 (−1.99, 3.19)	−0.58 (−3.03, 1.87)	−2.26 (−4.85, 0.34)
Number of reading books at age 7	0.62 (0.39, 0.84)	0.16 (−0.05, 0.38)	0.57 (0.35, 0.80)
Living with both parents at age 15			
Yes	3.46 (0.44, 6.49)	−0.41 (−3.27, 2.45)	−0.27 (−3.30, 2.75)
Equivalent income at age 15			
Low	ref.	ref.	ref.
Lower	−0.30 (−2.84, 2.25)	3.40 (0.99, 5.81)	2.40 (−0.15, 4.95)
Higher	−0.23 (−2.83, 2.38)	4.81 (2.34, 7.28)	3.40 (0.79, 6.01)
Highest	−0.45 (−3.27, 2.38)	6.79 (4.12, 9.47)	2.90 (0.07, 5.73)
Size of residential city at age 15			
Large city	ref.	ref.	ref.
Middle city	0.36 (−2.46, 3.18)	−0.08 (−2.82, 2.66)	0.56 (−2.36, 3.48)
Small city	−0.29 (−2.79, 2.21)	−1.45 (−3.88, 0.97)	−0.09 (−2.65, 2.46)
Town	−2.16 (−5.80, 1.48)	0.10 (−3.39, 3.58)	1.26 (−2.42, 4.93)
**Random effects**			
Prefecture-level variance	0.03 (−0.02, 0.09)	0.05 (−0.01, 0.11)	0.02 (−0.03, 0.06)
City-level variance	0.05 (−0.09, 0.20)	0.08 (−0.06, 0.22)	0.17 (−0.00, 0.33)
Individual-level variance	23.82 (23.21, 24.44)	21.27 (20.72, 21.82)	23.77 (23.15, 24.38)

All listed variables were simultaneously included in the models.
Missing categories for covariates were included in the analysis but not reported to reduce the space

Supplementary Table S4. Stratification analysis by equivalent income.

	Equivalent income strata			
	Lowest	Lower	Higher	Highest
	Coef. (95% CI)	Coef. (95% CI)	Coef. (95% CI)	Coef. (95% CI)
**Intrinsic motivation**				
Fixed effects				
Library density				
Low	ref.	ref.	ref.	ref.
Middle	4.83 (0.05, 9.60)	−0.36 (−5.38, 4.67)	−0.49 (−5.55, 4.56)	2.61 (−3.32, 8.54)
High	8.60 (3.41, 13.78)	2.45 (−2.88, 7.78)	0.86 (−4.23, 5.95)	2.39 (−3.46, 8.23)
Random effects				
Prefecture-level variance	0.04 (−0.11, 0.20)	0.05 (−0.11, 0.21)	0.00 (0.00, 0.00)	0.00 (0.00, 0.00)
City-level variance	0.00 (0.00, 0.00)	0.36 (−0.28, 1.00)	0.00 (0.00, 0.00)	0.00 (0.00, 0.00)
Individual-level variance	23.47 (22.27, 24.68)	23.27 (21.95, 24.59)	23.88 (22.69, 25.07)	24.12 (22.86, 25.38)
**Extrinsic motivation**				
Fixed effects				
Library density				
Low	ref.	ref.	ref.	ref.
Middle	2.72 (−1.63, 7.08)	−1.17 (−5.74, 3.40)	−3.15 (−8.02, 1.71)	−1.83 (−7.64, 3.98)
High	3.32 (−1.34, 7.98)	−4.12 (−8.90, 0.66)	0.55 (−4.38, 5.49)	−3.47 (−9.20, 2.26)
Random effects				
Prefecture-level variance	0.00 (0.00, 0.00)	0.00 (0.00, 0.00)	0.02 (−0.09, 0.13)	0.00 (0.00, 0.00)
City-level variance	0.00 (0.00, 0.00)	0.00 (0.00, 0.00)	0.00 (0.00, 0.00)	0.10 (−0.38, 0.58)
Individual-level variance	19.74 (18.73, 20.75)	21.26 (20.19, 22.34)	21.89 (20.79, 22.98)	22.58 (21.32, 23.85)
**Altruistic motivation**				
Fixed effects				
Library density				
Low	ref.	ref.	ref.	ref.
Middle	4.22 (−0.52, 8.95)	−2.47 (−7.34, 2.41)	4.50 (−0.80, 9.81)	1.09 (−4.94, 7.12)
High	1.97 (−3.10, 7.04)	−3.15 (−8.34, 2.04)	2.32 (−3.10, 7.73)	−2.50 (−8.44, 3.45)
Random effects				
Prefecture-level variance	0.00 (0.00, 0.00)	0.05 (−0.10, 0.21)	0.04 (−0.11, 0.19)	0.00 (0.00, 0.00)
City-level variance	0.00 (0.00, 0.00)	0.00 (0.00, 0.00)	0.41 (−0.19, 1.01)	0.14 (−0.38, 0.66)
Individual-level variance	23.34 (22.15, 24.53)	23.79 (22.58, 25.00)	23.45 (22.16, 24.73)	24.09 (22.74, 25.45)

Abbreviations: coefficient, Coef; confidence interval, CI

Models were adjusted for child's gender, mother's age, father's age, parents' education, having older siblings, having younger siblings, either self-employment, living with both parents, and size of municipality of residence.

Supplementary Table S5. Stratification analysis by size of municipality of residence at age 15.

	Size of residential city at age 15	
	Major/Middle city	Small city/Town
	Coef. (95% CI)	Coef. (95% CI)
**Intrinsic motivation**		
Fixed effects		
Library density		
Low	ref.	ref.
Middle	2.14 (−2.35, 6.63)	1.63 (−1.60, 4.86)
High	4.37 (−0.17, 8.91)	2.67 (−0.74, 6.07)
Random effects		
Prefecture-level variance	0.07 (−0.04, 0.18)	0.03 (−0.05, 0.11)
City-level variance	0.06 (−0.13, 0.26)	0.11 (−0.20, 0.42)
Individual-level variance	23.82 (22.95, 24.69)	23.63 (22.75, 24.51)
**Extrinsic motivation**		
Fixed effects		
Library density		
Low	ref.	ref.
Middle	−1.08 (−5.33, 3.16)	−0.41 (−3.48, 2.67)
High	−2.07 (−6.35, 2.20)	0.77 (−2.53, 4.07)
Random effects		
Prefecture-level variance	0.05 (−0.04, 0.14)	0.06 (−0.03, 0.15)
City-level variance	0.06 (−0.11, 0.24)	0.12 (−0.16, 0.40)
Individual-level variance	21.56 (20.77, 22.35)	20.92 (20.14, 21.70)
**Altruistic motivation**		
Fixed effects		
Library density		
Low	ref.	ref.
Middle	−0.39 (−5.02, 4.25)	3.16 (−0.16, 6.48)
High	−2.77 (−7.36, 1.82)	1.63 (−1.94, 5.20)
Random effects		
Prefecture-level variance	0.02 (−0.06, 0.10)	0.07 (−0.03, 0.18)
City-level variance	0.17 (−0.04, 0.38)	0.22 (−0.11, 0.55)
Individual-level variance	23.75 (22.88, 24.62)	23.58 (22.70, 24.46)

Abbreviations: coefficient, Coef; confidence interval, CI

Models were adjusted for child's gender, mother's age, father's age, parents' education, having older sibling, having younger sibling, either self-employment, living with both parents, and equivalent income.

Supplementary Figure S1. Flowchart for the study participants

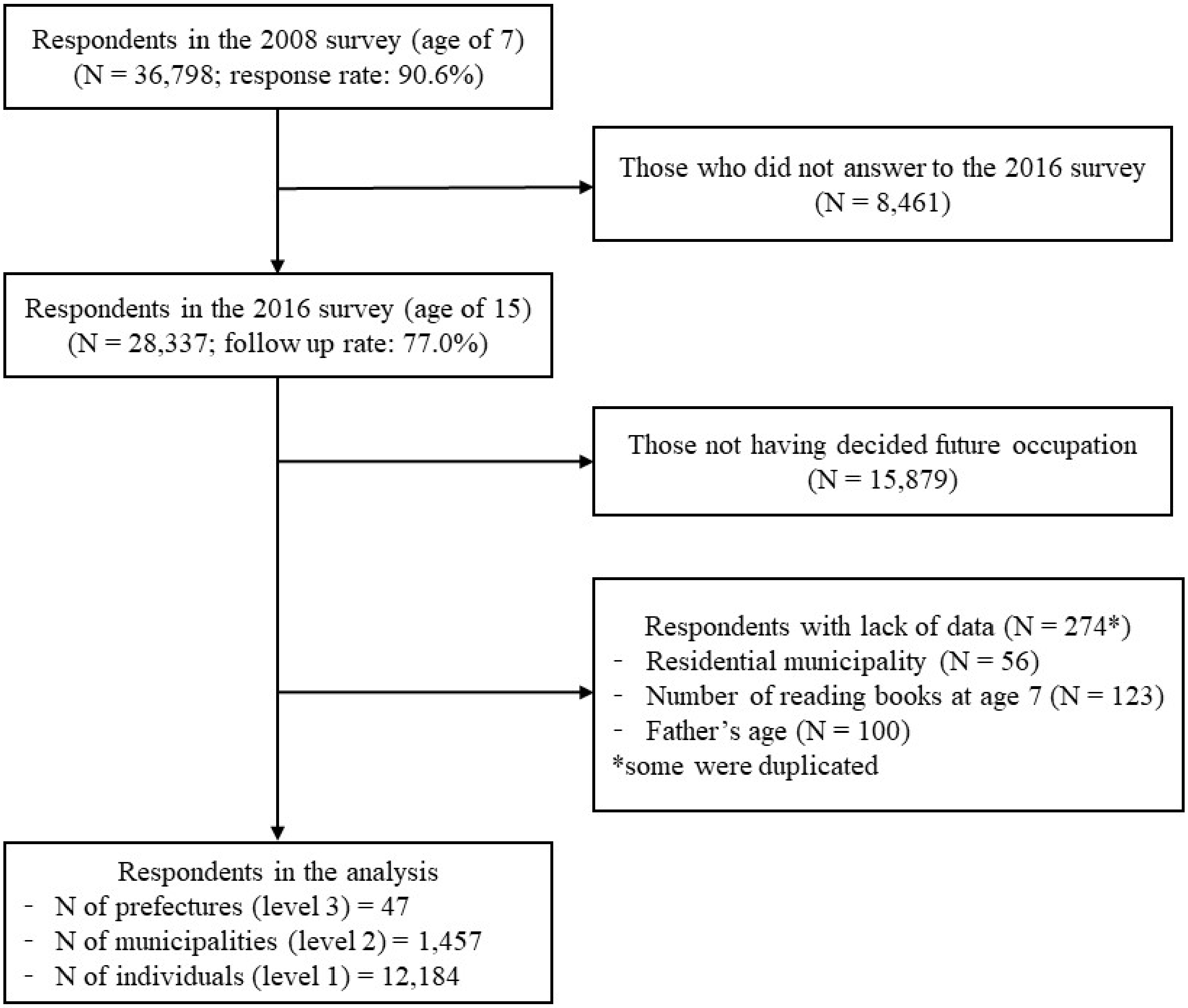

Supplement: Supplementary file 1 [file ijerph-18-11209-s001.zip › ijerph-1358377-supplementary.pdf]
